# Seroprevalence of *Toxoplasma gondii* in Dairy Cattle with Reproductive Problems in Sudan

**DOI:** 10.1155/2013/895165

**Published:** 2013-09-19

**Authors:** Abdelghafar M. Elfahal, Amira M. Elhassan, Mohammed O. Hussien, Khalid A. Enan, Azza B. Musa, Abdelrahim M. El Hussein

**Affiliations:** ^1^Central Laboratory, Ministry of Science and Technology, P.O. Box 7099, Khartoum, Sudan; ^2^Veterinary Research Institute, P.O. Box 8067, Khartoum, Sudan; ^3^Animal Resources, Research Corporation, P.O. Box 8067, Khartoum, Sudan

## Abstract

Toxoplasmosis, caused by *Toxoplasma gondii*, is one of the most common parasitic infections of humans and other warm-blooded animals in most parts of the world. The disease is common among sheep and goats and it is recognized as one of the major causes of reproductive failure in these animals. Cattle, on the other hand, can be infected, but abortion or perinatal mortality has not been recorded. This survey was carried out to study the prevalence of this disease in cattle in Khartoum and Gazira States (Sudan). 181 sera samples collected from dairy cattle with reproductive problems were assayed for antibodies to *T. gondii* by ELISA. The prevalence rate of *T. gondii* antibodies in cattle at herd level was 44.8% (13/29). Herd level infection rates were 50% and 33.3% in Khartoum and Gazira States, respectively. The overall prevalence of *T. gondii* at individual level in both states was 13.3% (24/181). The prevalence was 12.7% (17/134), was 14.9% (7/47) in Khartoum and Gazira State, respectively. There was significantly higher (*P* < 0.05) prevalence of *T. gondii* antibodies in the age group less than one year old (36.4%) than in other age groups and in males (30.8%) than in females (11.9%) while no significant relationship was discerned regarding breed, location, season, or signs of reproductive disease.

## 1. Introduction

Toxoplasmosis is one of the most common human parasitic infections worldwide with an estimated prevalence in 1-2 billion people [1]. Toxoplasmosis is a zoonotic disease caused by a sporozoan parasite, *Toxoplasma gondii*, distributed throughout the world. The definitive hosts are domestic cats and various species of wild felids and the intermediate hosts are mammals and birds [2].

Transmission occurs following ingestion of sporulated oocysts or bradyzoites within cysts present in the tissues of numerous food animals. The frequency of infection is extremely variable in the different regions of the world. Seroprevalence in the human population reportedly ranges from 0 to 90% [3] and infection is more common in warm climates and in low-lying areas than in cold climates and mountainous regions, where conditions for sporulation and survival of oocysts are less favourable [4]. The prevalence of *T. gondii *infection also varies between ethnic groups, and it is thought that this is largely due to sanitary and cooking habits rather than genetic differences. A seroprevalence of 80% has been reported from Paris where undercooked meat is often consumed [4]. Lower seroprevalences (10–40%) have been reported in countries from Southeast Asia where meat is cooked thoroughly [5]. 

There is limited evidence supporting the hypothesis that cattle are not a favoured host for *T. gondii *but in general infection is more likely to occur from consumption of pork, lamb's and goat's meat than from beef [6–8] and consumption of cured pork has indeed been recognized as an important source of *T. gondii* infection [9].

Limited information is available on the prevalence of toxoplasmosis in man and animals in Sudan. Camel toxoplasmosis was first reported in Sudan by El Din et al. [10] who recorded an infection rate of 54%. Afterwards Bornstein and Musa [11] and Elamin et al. [12] reported 22.5% and 11.8% in Sudanese camels, respectively. A more recent study recorded prevalence rates of toxoplasma antibodies of 20%, 32%, and 57.5% in camels, cattle, and sheep, respectively [13]. Prevalence rates of up to 73% were recorded in childbearing age women and up to 100% in camel herders in Sudan [14–17]. 

The present study was carried out to assay seroprevalence of *T. gondii* in dairy cattle herds with reproductive problems and to determine risk factors associated with the infection in two states, namely, Khartoum and Gazira of Sudan. 

## 2. Materials and Methods

A total of 181 (168 females and 13 males) serum samples were taken from dairy cattle herds with reproductive problems such as abortion, infertility, and stillbirth. Dairy herds were raised under semi-intensive husbandry systems in farms around Khartoum State (Khartoum, Khartoum North, and Omdurman) and Gazira States (Al Kamleen and Wad Madani). Blood was collected from cattle by vein puncture of the jugular vein. Sera were harvested following centrifugation of clotted blood, labeled, and stored at −20°C until tested. Antibodies to *T. gondii* were tested by indirect enzyme linked imunosorbent assay (iELISA). A herd was considered positive for *T. gondii *when at least one animal with positive antibody reaction was detected. 

### 2.1. ELISA Technique

Commercial iELISA kits (Ruminant Serum Toxoplasmosis) for detection of anti*-T. gondii* antibodies were purchased from Lsivet (Nouzilly, France). Positive serum samples will present yellow colour; the colour visualized in each well is proportional to the titer of antibody specific to *T. gondii* present in the diluted sample (1/400). All samples which have antibody titer ≥20 are considered positive. 

### 2.2. Statistical Analysis

The serological results and other information gathered during this investigation such as season, location, breed, sex, and age of the sampled animals were edited and analyzed statistically using statistical package (SPSS version 13). To identify the association of the risk factors with the specific seroprevalence, the chi-square (*χ* ^2^ test) and one-way ANOVA were used. The statistical significance level used was *P* ≤ 0.05.

## 3. Results

### 3.1. Herd Infection Level

Out of 29 herds tested at both states, 13 (44.8%) proved to be positive for *T. gondii* antibodies by ELISA. The result showed that 10 (50%) out of the 20 herds in Khartoum State and 3 (33.3%) out of nine herds in Gazira State were positive with the highest herd prevalence (57.1%) being detected in Khartoum North locality (Khartoum State) (Table 1).

### 3.2. Individual Infection Level

One hundred and eighty one serum samples from Khartoum and Gazira States were assayed for the presence of anti-*Toxoplasma gondii* antibodies using ELISA. The overall prevalence of *T. gondii* in both states was 13.3% (24/181). The prevalence rate was 12.7% (17/134) in Khartoum State and the prevalence according to the locality was as follows: 12.9% (8/62), 14% (6/43), and 10.3% (3/29) were recorded for Khartoum, Khartoum North and Omdurman localities, respectively. In Gazira State the prevalence rate was 14.9% (7/47) and positive samples were detected in Alkamleen 25% (2/8) and Wad Madani 12.8% (5/39) localities (Table 2). 

The prevalence of *T. gondii *infection was significantly (*P* < 0.05) higher (36.4%) in animals less than one year old than those above two years (12.8%). Moreover, significantly (*P* < 0.05) higher seroprevalence of *T. gondii* was observed in males (30.8%) than in females (11.9%), but there was no significant relationship between antibodies prevalence and breed, location, season, or signs of reproductive disease (Table 3). 

## 4. Discussion

Until now insufficient data are available on cattle toxoplasmosis in the world, and there have been limited number of reports on cattle toxoplasmosis from Sudan. The overall seroprevalence of toxoplasmosis reported in the present study in Sudan using ELISA (13.3%) is higher than that reported for cattle in China (2.3%) [18] and Vietnam (10.5%) [19] but is comparable to that reported in Iran (15.91%) [2] and lower than that (27.9%) recorded in The Netherlands [20]. The differences in prevalence rates between countries may be due to the samples size of different studies besides the different techniques used. However, data on the seroprevalence in cattle show great variation, ranging from 0 to 99% [21, 22]; hence, such data should be analyzed with caution as geographical variations occur not only among different countries but also within countries.

In the present study* T. gondii* antibodies were prevalent both at herd and at individual animal levels. Out of the 20 herds tested in Khartoum State, nine (45%) herds proved to be positive for *T. gondii* antibodies and three (33.3%) of nine herds in Gazira State were positive. The highest infection rate (57.1%) among herds was observed in Khartoum North in Khartoum State. Although no statistically significant association was found regarding signs of reproductive disease, this high prevalence rate (four herds out of seven were infected) is in concordance with high abortion rates reported from farms in this particular area.

The seroprevalence of *T. gondii* infection reported in the current study in Khartoum State was 13.3% using ELISA. Khalil and Intisar [13] reported that the prevalence rate of anti*-T. gondii* antibodies in cattle in Khartoum State using latex agglutination test (LAT) was 32%. The difference in the prevalences between the two studies could be attributed to the different techniques used in estimating these prevalences and to the lower number (50) of cattle investigated by Khalil and Intisar [13]. Positive cases were more frequent among cattle less than one year old (36.4%, *P* < 0.05) than cattle above two years old. This is in concordance with Nematollahi and Moghddam [2] who reported similar results in Iran in 2008. This may reflect dominance of maternally acquired antibodies in this age group or that the cattle unless reinfected, deplete antibodies as their age increases [3]. Cattle are generally described as insensitive to *T. gondii* infection. Cattle would harbour few parasite tissue cysts, which may not persist for the lifetime of the host [23–25].

The prevalence was also observed to be significantly (*P* ≤ 0.05) higher in males (30.8%) than females. Similar results were obtained by Nematollahi and Moghddam [2]. However, the numbers of males assayed for *T. gondii* antibodies in the present study were too low (13) to allow for any concrete conclusions regarding association of gender with the seroprevalence of this parasite.

It could be concluded that toxoplasmosis is prevalent in dairy herds in Khartoum and Gazira States. Age and gender were observed to be the main risk factors involved, while no association with signs of reproductive disease such as abortion, stillbirth, and infertility was discerned. The results also suggest that toxoplasma oocysts and reservoir host are widely distributed in the environment. Hence, further epizootiological and parasitological investigations on toxoplasmosis in cattle and other farm animals and human at the country level are required to determine the magnitude of the infection and to estimate its economic impact; the relative importance of different species in the epidemiology of toxoplasmosis; and its public health hazard in Sudan.

## Figures and Tables

**Table 1:** Herd level prevalence of *T. gondii* antibodies in cattle in Khartoum and Gazira States detected by ELISA.

Area	Location	No. of herds	Total no. of animals	+ve herds	+ve%
Khartoum	Khartoum	8	240	4	50
	Khartoum North	7	250	4	57.1
	Omdurman	5	210	2	40
Subtotal		20		10	50%

Gazira	Alkamleen	1	30	1	100
	Wad Madani	8	370	2	25
Subtotal		9		3	33.3

Total		29	1100	13	44.8

**Table 2:** Seropositivity to *T. gondii* in cattle from different locations in Khartoum State and Gazira State detected by ELISA.

Area	Location	No. tested	Positive	Positive %
Khartoum	Khartoum	62	8	12.9
	Khartoum North	43	6	14
	Omdurman	29	3	10.3
Subtotal		134	17	12.7

Gazira	Alkamleen	8	2	25
	Wad Madani	39	5	12.8
Subtotal		47	7	14.9

Total		181	24	13.3

**Table 3:** Influence of some risk factors on seroprevalence of *T. gondii* in cattle using ELISA in Khartoum and Gazira States.

Variable	Groups	No. tested	Positive	Positive %
Age (year)	<1	11	4	36.4*
	1-2	14	0	0.00
	>2	156	20	12.8
Sex	Male	13	4	30.8*
	Female	168	20	11.9
Breed	Cross	175	23	13.1
	Local	6	1	16.7
Location	Khartoum	134	17	12.7
	Gazira	47	7	14.9
Seasons	Winter	90	11	12.2
	Dry	26	6	23.1
	Rainy	65	7	10.8
Health status	Apparently healthy	75	9	12.0
	Abortion	63	12	19.0
	Infertility	27	1	3.7
	Death of calf after birth	16	2	12.5

*Significant (*P* ≤ 0.05).
